# Supplementary material for: Rank- and threat-dependent social modulation of innate defensive behaviors
Source: eLife. 2026 Sep 15;15:RP109571. doi: 10.7554/eLife.109571 (PMC13577663; doi:10.7554/eLife.109571)
Supplement: Peer review history

# Editor report: eLife Assessment

**DOI:** 10.7554/eLife.109571.3.sa0

**Authors:** Peng Cao

**Affiliations:** National Institute of Biological Sciences, Beijing China

**Keywords:** Convincing, Important

The authors investigate how dominance hierarchy shapes defensive strategies in mice under two naturalistic threats: a transient visual looming stimulus and a sustained live rat. This study provides **important** insights into how social context and dominance hierarchy modulate innate defensive behaviors across distinct naturalistic threats. The strength of evidence is **convincing**, with detailed classification and analysis of behaviors.

# Referee report: Reviewer #1 (Public review)

**DOI:** 10.7554/eLife.109571.3.sa1

Summary:

This study presents an interesting behavioral paradigm and reveals interactive effects of social hierarchy and threat type on defensive behaviors. However, addressing the aforementioned points regarding methodological detail, rigor in behavioral classification, depth of result interpretation, and focus of the discussion is essential to strengthen the reliability and impact of the conclusions in a revised manuscript.

Strengths:

The paper is logically sound, featuring detailed classification and analysis of behaviors, with a focus on behavioral categories and transitions, thereby establishing a relatively robust research framework.

Comments on revised version.

I think the authors have addressed all of my comments.

# Referee report: Reviewer #2 (Public review)

**DOI:** 10.7554/eLife.109571.3.sa2

Summary

The authors examine how dominance hierarchy modulates defensive strategies in mice exposed to two naturalistic threats: a transient visual looming stimulus and a sustained live rat. By comparing single versus paired testing conditions, they demonstrate that social presence attenuates fear responses, and that dominant and subordinate mice display distinct behavioral and social patterns depending on threat type. The study offers a rich behavioral dataset and a potentially valuable framework for investigating hierarchical influences on innate fear.

Strengths

(1) The use of two ecologically relevant threat paradigms allows for meaningful comparisons across transient and sustained contexts.

(2) Behavioral quantification is thorough, incorporating manual annotation of multiple behavior types and transition‑matrix analyses.

(3) The comparison between dominant and subordinate pairs is novel within the innate‑fear literature.

(5) The manuscript is well structured and clearly written, with figures that are visually informative and effectively support the main conclusions.

Weaknesses

The investigation of neural mechanisms underlying the observed behavioral effects remains limited.

# Referee report: Reviewer #3 (Public review)

**DOI:** 10.7554/eLife.109571.3.sa3

Summary:

This study examines how dominance hierarchy influences innate defensive behaviors in pair-housed male mice exposed to two types of naturalistic threats: a transient looming stimulus and a sustained live rat. The authors show that social presence reduces fear-related behaviors and promotes active defense, with dominant mice benefiting more prominently. They also demonstrate that threat exposure reinforces social roles and increases group cohesion. The work highlights the bidirectional interaction between social structure and defensive behavior.

Strengths:

This study makes a valuable contribution to behavioral neuroscience through its well-designed examination of socially modulated fear. A key strength is the use of two ethologically relevant threat paradigms - a transient looming stimulus and a sustained live predator, enabling a nuanced comparison of defensive behaviors. The experimental design is robust, systematically comparing animals tested alone versus with their cage mate to cleanly isolate social effects. The behavioral analysis is sophisticated, employing detailed transition maps that reveal how social context reshapes behavioral sequences, going beyond simple duration measurements. The finding that social modulation is rank-dependent adds significant depth, linking social hierarchy to adaptive defense strategies. Furthermore, the demonstration that threat exposure reciprocally enhances social cohesion provides a compelling systems-level perspective. Together, these elements establish a strong behavioral framework for future investigations into the neural circuits underlying socially modulated innate fear.

Comments on revised version.

The authors have addressed the initial major criticism regarding the lack of causal evidence for neural mechanisms, which has alleviated our concerns. This provides a more solid behavioral foundation for future investigations into neural circuit mechanisms.

# Author comment: Author response

**DOI:** 10.7554/eLife.109571.3.sa4

**Authors:** Ling-yun Li, Xinjian Gao, Jun Zhang, Wen-wei Wu, Ya-tang Li

**Affiliations:** Capital Medical University Beijing China; Chinese Institute for Brain Research Beijing China; Capital Medical University Beijing China; Capital Medical University Beijing China; Chinese Institute for Brain Research Beijing China

The following is the authors’ response to the original reviews.

**Public Reviews:**

**Reviewer #1 (Public review):**

Summary:

This study presents an interesting behavioral paradigm and reveals interactive effects of social hierarchy and threat type on defensive behaviors. However, addressing the aforementioned points regarding methodological detail, rigor in behavioral classification, depth of result interpretation, and focus of the discussion is essential to strengthen the reliability and impact of the conclusions in a revised manuscript.

Strengths:

The paper is logically sound, featuring detailed classification and analysis of behaviors, with a focus on behavioral categories and transitions, thereby establishing a relatively robust research framework.

Weaknesses:

Several points require clarification or further revision.

(1) Methods and Terminology Regarding Social Hierarchy:

The study uses the tube test to determine subordinate status, but the methodological description is quite brief. Please provide a more detailed account of the experimental procedure and the criteria used for determination.

We have included more details about how the tube test was performed in the revised manuscript. Social rank within each mouse pair was determined using a standard tube test paradigm. To minimize stress, mice were pair-housed for at least two weeks with a 15-cm tube placed in their home cage to allow voluntary exploration. Prior to rank assessment, mice were trained to traverse a 30-cm tube over two consecutive days (10 trials per day), with alternating entry from either end to prevent side bias. On the test day, each mouse pair underwent up to seven competitive trials in the same 30-cm tube. In each trial, the two mice were simultaneously released from opposite ends of the tube. A “win” was defined as one mouse successfully advancing through the tube while the opponent retreated completely out of the tube (all four paws outside) for at least 5 seconds. The first mouse to achieve four wins was designated as the dominant individual, whereas the opponent was classified as subordinate. Social rank stability was reassessed one day after threat exposure using the same criteria. Only pairs with consistent ranks were included in subsequent analyses.

The dominance hierarchy is established based on pairs of mice. However, the use of terms like "group cohesion" - typically applied to larger groups - to describe dyadic interactions seems overstated. Please revise the terminology to more accurately reflect the pairwise experimental setup.

Thanks for the comment. We have replaced the term “group cohesion” with “social engagement”.

(2) Criteria and Validity of Behavioral Classification:

The criteria for classifying mouse behaviors (e.g., passive defense, active defense) are not sufficiently clear. Please explicitly state the operational definitions and distinguishing features for each behavioral category.

Passive defense was defined as an immobility-based defensive strategy characterized by suppression of locomotor activity, including freezing and tail rattling. Active defense was defined as movement- or posture-dependent defensive strategy, including approach, investigation, withdrawal, and stretch-attend. We have clarified these in the revised manuscript.

How was the meaningfulness and distinctness of these behavioral categories ensured to avoid overlap? For instance, based on Figure 3E, is "active defense" synonymous with "investigative defense," involving movement to the near region followed by return to the far region? This requires clearer delineation.

Defensive behaviors in the rat exposure paradigm were grouped into two categories: passive and active defense, each comprising distinct behaviors. All the manually annotated behaviors were mutually exclusive; that is, each video frame was assigned a single behavioral label to avoid overlap across behaviors. Active defense includes four behaviors: approach, investigation, withdrawal, and stretch-attend. We have clarified these points in the revised manuscript.

The current analysis focuses on a few core behaviors, while other recorded behaviors appear less relevant. Please clarify the principles for selecting or categorizing all recorded behaviors.

Thank you for pointing this out. In the current study, we focused primarily on defensive and social behaviors. We also included several neutral solitary behaviors related to anxiety and defensive state, such as sniffing, grooming, and rearing, which were consistently expressed across animals and closely linked to our main findings. We have clarified these in the revised manuscript.

(3) Interpretation of Key Findings and Mechanistic Insights:

Looming exposure increased the proportion of proactive bouts in the dominant zone but decreased it in the subordinate zone (Figure 4G), with a similar trend during rat exposure. Please provide a potential explanation for this consistent pattern. Does this consistency arise from shared neural mechanisms, or do different behavioral strategies converge to produce similar outputs under both threats?

Thanks for bringing up this important question. The consistent increase in proactive bouts in dominant mice across both paradigms suggests a consistent rank-dependent reorganization of dyadic interaction under threats. We propose that this convergence reflect a shared neural mechanism that links defensive state with social-rank information, potentially involving top-down regulation from the mPFC to threat-specific midbrain and hypothalamic defensive circuits. We have expanded the discussion to incorporate this explanation.

(4) Support for Claims and Study Limitations:

The manuscript states that this work addresses a gap by showing defensive responses are jointly shaped by threat type and social rank, emphasizing survival-critical behaviors over fear or stress alone. However, it is possible that the behavioral differences stem from varying degrees of danger perception rather than purely strategic choices. This warrants a clear description and a deeper discussion to address this possibility.

We thank the reviewer for this insightful comment. We agree that, in principle, behavioral differences could arise from variations in perceived danger rather than strategic choice. In humans, decisions can sometimes reflect value-based strategies that override perceived danger. In contrast, under naturalistic threat conditions, mice likely rely predominantly on danger perception to make behavioral decisions, and such responses are expected to be consistent with value-based strategies shaped by natural selection. In the revised manuscript, we have expanded the Discussion to address the role of threat perception and its relationship to decision-making in our behavioral paradigms.

The Discussion section proposes numerous brain regions potentially involved in fear and social regulation. As this is a behavioral study, the extensive speculation on specific neural circuitry involvement, without supporting neuroscience data, appears insufficiently grounded and somewhat vague. It is recommended to focus the discussion more on the implications of the behavioral findings themselves or to explicitly frame these neural hypotheses as directions for future research.

We have revised the Discussion to focus more directly on behavioral findings and added explicit neural hypotheses as potential future directions.

**Reviewer #2 (Public review):**

Summary:

The authors investigate how dominance hierarchy shapes defensive strategies in mice under two naturalistic threats: a transient visual looming stimulus and a sustained live rat. By comparing single versus paired testing, they report that social presence attenuates fear and that dominant and subordinate mice exhibit different patterns of defensive and social behaviors depending on threat type. The work provides a rich behavioral dataset and a potentially useful framework for studying hierarchical modulation of innate fear.

Strengths:

(1) The study uses two ecologically meaningful threat paradigms, allowing comparison across transient and sustained threat contexts.

(2) Behavioral quantification is detailed, with manual annotation of multiple behavior types and transition-matrix level analysis.

(3) The comparison of dominant versus subordinate pairs is novel in the context of innate fear.

(4) The manuscript is well-organized and clearly written.

(5) Figures are visually informative and support major claims.

Weaknesses:

Lack of neural mechanism insights.

The current study focused on behavior. In the revised manuscript, we have incorporated a discussion of potential neural mechanisms and highlight this as an important direction for future work.

**Reviewer #3 (Public review):**

Summary:

This study examines how dominance hierarchy influences innate defensive behaviors in pair-housed male mice exposed to two types of naturalistic threats: a transient looming stimulus and a sustained live rat. The authors show that social presence reduces fear-related behaviors and promotes active defense, with dominant mice benefiting more prominently. They also demonstrate that threat exposure reinforces social roles and increases group cohesion. The work highlights the bidirectional interaction between social structure and defensive behavior.

Strengths:

This study makes a valuable contribution to behavioral neuroscience through its well-designed examination of socially modulated fear. A key strength is the use of two ethologically relevant threat paradigms - a transient looming stimulus and a sustained live predator, enabling a nuanced comparison of defensive behaviors. The experimental design is robust, systematically comparing animals tested alone versus with their cage mate to cleanly isolate social effects. The behavioral analysis is sophisticated, employing detailed transition maps that reveal how social context reshapes behavioral sequences, going beyond simple duration measurements. The finding that social modulation is rank-dependent adds significant depth, linking social hierarchy to adaptive defense strategies. Furthermore, the demonstration that threat exposure reciprocally enhances social cohesion provides a compelling systems-level perspective. Together, these elements establish a strong behavioral framework for future investigations into the neural circuits underlying socially modulated innate fear.

Weaknesses:

The study exhibits several limitations. The neural mechanism proposed is speculative, as the study provides no causal evidence.

Establishing causal evidence for neural mechanisms is beyond the scope of the current behavioral study. We highlight this as an important direction for future work in the revised manuscript.

**Recommendations for the authors:**

**Reviewer #2 (Recommendations for the authors):**

(1) Clarify the definitions of all behavioral categories (escape, assessment, passive defense, active defense, etc.) in detail.

We have clarified the definitions of all behavioral categories in detail in the revised manuscript.

(2) Reduce speculative statements about SC-VMHdm-mPFC circuitry, and provide the data about this circuit, if possible.

We have reduced the speculative statements about this circuitry in the revised manuscript.

**Reviewer #3 (Recommendations for the authors):**

We commend the authors on a carefully executed and conceptually clear study that makes a valuable contribution to behavioral neuroscience. Below are some issues and suggestions for this manuscript:

(1) Please add the following to the discussion: the reason why, compared to looming exposure, social behaviors were more often followed by defensive behavior during rat exposure.

We have added this discussion to the revised manuscript. This difference reflects the distinct temporal characteristics of the two threats. Following the transient looming stimulus, the threat rapidly ceases once the stimulus ends, reducing the need for sustained defensive behavior. Consequently, social interactions primarily occur after threat termination and are less frequently interleaved with subsequent defensive behaviors. Consistent with this interpretation, looming exposure selectively increased the duration of social behavior in subordinate mice. In contrast, rat exposure represents a sustained multisensory threat that maintains a persistently elevated defensive state. Under these conditions, both the frequency and duration of social interactions increased in dominant and subordinate mice. Moreover, huddling emerged as the predominant social behavior, and the frequent transitions between freezing and huddling suggest that social interactions become integrated with ongoing defensive responses, potentially serving as a safety-seeking or cohesive defense during sustained threat.

(2) Figures 1B and 1C showed that Grooming has significantly decreased. Please verify whether the statistical methods and results are correct.

The grooming data in the original Figure 1C did not distinguish dominant and subordinate mice and did not show significant decrease. We have removed it in the revised manuscript. In original Figure 2I (current Figure 1P), the social modulation on grooming behavior was observed only in dominant mice (Two-way ANOVA with post hoc Tukey’s range test).

(3) To investigate how social context modulates the expression and progression of defensive responses, the authors analyzed behaviors in two time windows: the early phase (0-5 seconds after stimulus onset) and the late phase (20-60 seconds after onset) in Figure2. What is the rationale for selecting 5 seconds as the cutoff for the early-phase behavioral analysis? Were the behaviors of mice between 5 and 20 seconds also analyzed?

The reason to select 5 seconds as the cutoff for early-phase behavioral analysis is that most behavioral decisions are made within this time window. We also analyzed the behaviors of mice between 5 and 20 seconds and have integrated them into the revised manuscript.

(4) Figure 2 demonstrated that the social context attenuates looming-evoked defensive behavior in a rank-dependent manner. Then, I would like to ask if the influence of the social context on rat-evoked defensive behavior is also present in a rank-dependent manner? Discuss the similarities and differences between the social context's effect on looming-evoked defensive behavior and rat behavior.

The influence of the social context on rat-evoked defensive behavior is also present in a rank-dependent manner. Specifically, total stretch-attend (SA) time and SA frequency were increased only in dominant mice (revised Figures 2Q and 2R), whereas average SA duration was decreased only in subordinate mice (revised Figure 2S). Approach-investigation-withdraw (AIW) frequency was increased only in dominant mice while approach speed was increased only in subordinate mice (revised Figures 2U and 2V).

Social context exerts a broad protective influence across threat types, but the form of this modulation differs depending on the nature of the threat. Similarity: social presence consistently alleviates threat-induced stress and reshapes defensive behavior in a rank-dependent manner, with dominant benefiting more strongly under both threats, suggesting higher social rank may associate with greater flexibility to integrate social modulation. Difference: the behavioral outcomes of social regulation are distinct across threat types. During looming, social presence primarily suppresses immediate defensive responses and alleviates post-looming anxiety, suggesting under transient and unpredictable threat, social context dampens acute defense and facilitates behavioral recovery. In contrast, during the sustained rat exposure, social presence promotes a shift in defensive strategy from passive to active defense, rather than mere suppression of defensive output. These results suggest that social context flexibly adjusts defensive behavior according to ecological demands by reducing excessive defense and anxiety in response to transient looming and facilitating active coping when threatened by a sustained live predator. These discussions have been included to the revised manuscript.

(5) In Figure 4, looming exposure increased these two measures only in subordinate individuals, whereas rat exposure affected both ranks, indicating threat-specific modulation of social behaviors. The visual looming paradigm primarily simulates visual stimuli triggered by aerial predators, while the rat exposure stimulus involves not only visual cues but also other sensory inputs, such as olfactory signals for the experimental animals. This may lead to differences in the defensive behaviors of mice, particularly increasing the proportion of proactive behaviors in dominant individuals. If olfactory information transmission is blocked during rat exposure, can the behavioral phenotypes shown in Figure 4 still be observed?

The rationale for incorporating both looming and rat exposure paradigms was to mimic the distinct, ethologically relevant predator encounters by rodents in natural environments. As the reviewer pointed out, the multimodality nature of the rat threat may contribute to differences in the defensive behaviors displayed by mice. This point is also acknowledged in our manuscript, where we state that rat exposure “imposes prolonged stress and elicits a broader repertoire of defensive behaviors”.

We agree that systematically dissecting the contribution of specific sensory modalities (e.g., olfactory, visual, or auditory) to defensive behaviors during rat exposure is an interesting and important question. Based on prior literature, we speculate that olfactory cues likely play a major role. However, the main aim of the present study is to investigate how social regulation of defensive behaviors depends on dominance hierarchy and threat type, rather than isolating modality-specific sensory mechanisms. Within this framework, we preserved the multisensory features of the rat exposure to maintain its ecological validity and to emphasize its distinction from the visual-only looming threat. We thank the reviewer for raising this insightful question, but it is beyond the scope of current study. We will consider it as an important direction for future investigations.

(6) Discuss the potential mechanisms for the similarities and differences in the impact of the looming threat and the rat threat on cohesive behavior?

We have expanded the Discussion to address the potential neural mechanisms underlying both the similarities and differences in the effects of looming and rat threats on social cohesion. As discussed in response to issue (1), we propose that the behavioral differences between the two threats arise from their distinct nature. Here, we further discuss a potential circuit mechanism. Specifically, we propose that the mPFC serves as a common hub integrating social context and dominance-related information with threat processing, thereby contributing to the shared enhancement of social engagement under both threats. We further speculate that the distinct patterns of social behavior may arise from partially distinct defensive circuits. SC-centered visual threat circuits may facilitate rapid post-threat social engagement by recruiting vigilance-related networks, whereas VMHdm-centered predator-defense circuits may promote sustained social cohesion by engaging neural circuits that support coordinated coping during persistent defensive states. We emphasize that these are hypotheses, and that future studies combining circuit-level recordings and causal perturbations within the behavioral framework established here will be required to test them.
